# Chagas Disease, France

**DOI:** 10.3201/eid1404.070489

**Published:** 2008-04

**Authors:** François-Xavier Lescure, Ana Canestri, Hugues Melliez, Stéphane Jauréguiberry, Michel Develoux, Richard Dorent, Jean-Baptiste Guiard-Schmid, Philippe Bonnard, Faïza Ajana, Valeria Rolla, Yves Carlier, Frederick Gay, Marie-Hélène Elghouzzi, Martin Danis, Gilles Pialoux

**Affiliations:** * Hôpital Tenon AP-HP, Paris, France; † Hôpital La Pitié Salpétrière AP-HP, Paris, France; ‡ Le Centre Hospitalier de Tourcoing, Tourcoing, France; § Instituto de Pesquisa Clinica Evandro Chagas – Fiocruz, Rio de Janeiro, Brazil; ¶ Université libre de Bruxelles, Brussels, Belgium; # Etablissement Français du Sang d’lle de France, Paris, France

**Keywords:** Chagas disease, American trypanosomiasis, Trypanosoma cruzi, imported disease, France, Europe, transfusion, dispatch

## Abstract

Chagas disease (CD) is endemic to Latin America; its prevalence is highest in Bolivia. CD is sometimes seen in the United States and Canada among migrants from Latin America, whereas it is rare in Europe. We report 9 cases of imported CD in France from 2004 to 2006.

## Abstract 2

Chagas Disease, France

Nine cases of Chagas disease (CD), although rare in France, have been diagnosed in the country from 2004 through 2006 (Appendix Table). These included 1 case of acute Chagas myocarditis (ACM), 4 cases of chronic Chagas cardiomyopathy (CCC), and 4 cases of indeterminate chronic Chagas (ICC) (asymptomatic patients seropositive for *Trypanosoma cruzi*) (*1*). The ACM case involved an otherwise healthy 26-year-old woman who was hospitalized in September 2004 when she returned from a 2-month stay in French Guiana. Her symptoms included fever, headache, photophobia, intermittent chest pain, and arthromyalgia. Physical examination showed a typical Romaña sign, i.e., unilateral periorbital swelling (Figure). No abnormalities were found on clinical workup; blood smears and cultures were negative. Results of lumbar puncture, chest radiography, and echocardiography were also negative. The electrocardiogram (ECG) showed anterior ST-segment depression. A smear of a blister adjacent to the eye showing the Romaña sign yielded *T. cruzi* on direct examination. PCR was not performed. The patient was treated orally with benznidazole, 150 mg twice a day, and had a good clinical response. Benznidazole was discontinued after 7 weeks because peripheral neuropathy had developed. *T. cruzi* serologic results remained negative until 4 months after ACM, either because of a lack of sensitivity or because the patient was treated as soon as possible at the onset of symptoms.

**Figure:**
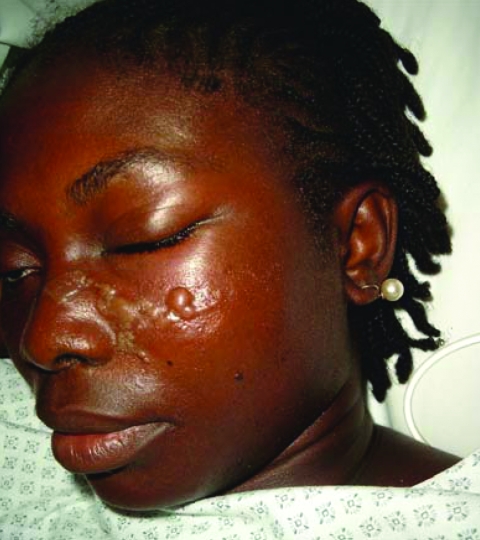
Romaña sign. Photo of female patient from French Guiana who lives in a metropolitan area of France. She had returned to Maripassoula to visit her parents during the holidays between July 13, 2004, and September 3, 2004. When the patient sought treatment on September 3, 2004, she had fever and unilateral periorbital edema.

The median age of the other 8 patients (4 men and 4 women from Bolivia) with chronic CD was 38 years (24–48). Seven patients had been living in France for 2 to 5 years. One of the patients with ICC was the son of a woman with CCC. Symptoms were mainly cardiologic, with atypical chest pain, dyspnea (New York Heart Association [NYHA] class 3–4), syncope, lipothymia, and fatigue (Appendix Table). Two patients were symptom free, including 1 in whom relatively severe cardiac disease was later diagnosed with. Five of these 8 patients had a family history of CCC. Clinically, all patients had bradycardia, hepatojugular reflux, or lower limb edema. Four patients had a normal clinical examination. No anomalies were found (complete blood cell count, transaminases, creatinine phosphokinase, troponin, C-reactive protein). The ECGs of all 4 patients with CCC showed bradycardia, including sinoatrial block (SAB) in 2 patients, and grade III atrioventricular block (AVB) in 2 patients. One patient had a right bundle branch block and a left anterior semiblock. Chest radiographs were normal. Transthoracic echocardiography showed a severe reduction in the left ventricular ejection fraction (20%) in 1 patient. Holter ECG confirmed the conduction abnormalities in 3 of the 4 patients (SAB, AVB, and ventricular hyperexcitability in 2 patients). All 8 patients had a positive indirect immunofluorescence test (IIF) and a positive ELISA test (*2*) for *T. cruzi* in serum (Appendix Table). The 8 patients were IIF-negative for *Leishmania* (*3*). The 2 patients with AVB III had pacemakers implanted and received angiotensin-converting-enzyme inhibitor and β-blocker therapy. Eight patients received oral benznidazole, 5 mg/kg/day for 1 to 8 weeks, depending on tolerability. Antihistamine therapy was given throughout benznidazole administration. One patient developed DRESS syndrome (drug rash with eosinophilia and systemic symptoms) after 2 weeks of treatment and improved a few days after benznidazole interruption. Nifurtimox was given (and was well tolerated) after the patient’s cutaneous and blood status had normalized. Three patients complained of numbness of the extremities during weeks 4, 5, and 7 of treatment; this pointed to benznidazole-induced peripheral neuropathies, which effectively disappeared when treatment was stopped. Three patients stopped taking their treatment prematurely; 1 patient was switched to nifurtimox after 2 weeks of treatment with benznidazole. Two patients reported a lessening of pain and improvements in their general health after antiparasitic treatment.

The prevalence of CD in *T.* *cruzi*–exposed, asymptomatic persons living in Europe is about 0.6% to 4% (*4**,**5*). Although CD remains extremely rare in Europe, a review of the literature shows 5 symptomatic cases up to 2004. There was 1 case of imported ACM in France in 1988 in a patient from Colombia (*6*), 1 case of autochthonous ACM in Spain in 1992 after blood transfusion (*7*), 1 case of imported CCC in Switzerland in 1996 in a patient from Bolivia (*8*), 1 case of imported ACM in Italy in 1997 in a patient from Brazil (*9*), and 1 case of imported ICC in Denmark in 2000 in a patient from Venezuela (*10*). After 2004, 3 additional cases were reported: 2 cases in Spain in 2005 (1 case of imported CCC in a patient from Bolivia) (*11*), 1 case of autochthonous neonatal ACM in the child of a Bolivian mother (*12*), and 1 case of imported CCC in the Netherlands in 2006 in a patient from South America (country not specified) (*13*).

Acute forms of CD diagnosed in Europe usually involve Europeans returning from stays in disease-endemic areas. The acute case described here underlines, as previously stated by Brisseau et al. (*6*), that a short stay in a disease-endemic zone, even for a few days, is sufficient to become a potential source of *T. cruzi*. In France, since April 2007, all persons who have spent any time in Central or South America are screened for *T. cruzi* before blood donation. This recent measure followed a series of 4 acute Chagas cases in French Guiana (*14*). Chronic imported forms usually involve South American immigrants, whose numbers are difficult to determine in Europe as many are illegal. The number of persons of Latin American origin living in metropolitan France has risen from 27,400 in 1999 to 105,000 in 2005 according to the National Institute for Demographic Studies (www.ined.fr). These persons are an underestimated potential source of transmission of disease. As illustrated by the cases recently reported by C. Riera (*12*), there is also a risk of transplacental transmission in women of South American origin living in Europe. CCC is sometimes life threatening, as in the case of patient 4 (Appendix Table), who had a very poor cardiac prognosis for a 38-year-old man.

The diagnosis of CD is not always straightforward in France. The current rarity of CD in Europe and the purely cardiologic (and sometimes gastrointestinal) manifestations of the chronic phase represent a diagnostic challenge. In France, few cardiologists and gastroenterologists are fully aware of this infectious disease. In the United States, because imported cases of CD are no longer exceptional, a Chagas screening test for blood donors was implemented in 2007 (*15*). The 9 cases we report, along with other recent cases, may be a sign that CD is emerging in France. If this imported disease becomes established in France, it could represent a real risk for transfusional and congenital transmission, not only in metropolitan areas in France but also in other European countries with a high Latin American immigrant population.

## Supplementary Material

Appendix Table Chagas disease cases reported in France since 2004*
