# Supplementary material for: Chagas Disease, France
Source: Emerg Infect Dis. 2008 Apr;14(4):644–9. doi: 10.3201/eid1404.070489 (PMC2570909; doi:10.3201/eid1404.070489)
Supplement: Appendix Table — Chagas disease cases reported in France since 2004* [file 07-0489_appT.pdf]

Appendix Table. Chagas disease cases reported in France since 2004*

Patient no.	Sex/ age	Year	Source	Type	Functional signs	Clinical signs	NYHA/ LVEF	ECG results	Serology and direct	Cardiac treatment	BNDZ duration, wk	Tolerability	Outcome
1	F/26	2004	French Guiana (Maripassoula)	ACM	Romaña, polyalgia	Hyperthermia	CI 1	Anterior ST-segment depression	TC in bulla (direct) IIF† 16	–	7	PN (wk 7)	Favorable
2	F/36	2004	Bolivia (Santa Cruz)	ICC	Asthenia, dyspnea, lipothymia	–	CI 3 70%	–	IIF‡ + 1600 ELISA§ + 4.6 (BM) 6.2 (BK)	–	–	Not relevant	Stable
3	F/40	2005	Bolivia (Santa Cruz)	CCC	Asthenia, abdominal and chest pain, lipothymia	Bradycardia, HJR, ELL	CI 2 65%	SAB, AVB3, VES	IIF¶ + 800 ELISA¶ + 5.2 (BM) 6.9 (BK)	PM, bisoprolol, perindopril	8	Fair	Favorable
4	M/38	2005	Bolivia (Santa Cruz)	CCC	Dyspnea, fainting	HJR, bradycardia	CI 4 20%	BAV3, VES, SAB	IIF§ + 400 ELISA¶ + 5.9 (BM) 6.6 (BK)	PM, bisoprolol, perindopril	8	PN (wk 6)	Favorable
5	M/48	2005	Bolivia (Santa Cruz)	ICC	Chest pain, striction	–	CI 1 70%	–	IIF§ + 400 ELISA¶ + 5.6 (BM) 6.8 (BK)	–	8	Fair	Favorable
6	M/38	2005	Bolivia (Santa Cruz)	CCC	Chest pain, lipothymia	Bradycardia	CI 1 70%	BBD, HBAG, VES	IIF§ + 1600 ELISA¶ + 5.2 (BM) 6.9 (BK)	–	4	PN (wk 4)	Favorable
7	F/42	2006	Bolivia (Santa Cruz)	CCC	Asthenia, polyalgia, constipation	Bradycardia	CI1 70%	Sinus bradycardi a	IF§ + 80 ELISA¶ + 0.57	–	8	PN (wk 8)	Favorable
8	M/24	2006	Bolivia (Santa Cruz)	ICC	Asymptomatic	–	CI1	–	IIF§ + 320 ELISA¶ + 1.88	–	8	Fair	Favorable
9	F/25	2006	Bolivia (Cochabamba)	ICC	Asymptomatic	–	CI1	–	IIF§ + 320 ELISA¶ + 1.46	–	2	Rash (wk 2)	Favorable

*NYHA , New York Heart Association classification; LVEF, left ventricular ejection fraction; ECG, electrocardiogram; wk, week; ACM, acute Chagas myocardiopathy; CI, confidence interval; TC, *Trypanosoma cruzi*; ICC, indeterminate chronic Chagas; CCC, chronic Chagas cardiomyopathy; HJR, hepatojugular reflux; ELL, edema of lower limbs; SAB, sinoatrial block; AVB, atrioentricular block; VES, ventricular extrasystoles; IIF, indirect Immunofluorescence; ELISA , enzyme linked immunosorbent assay; BM, Biomérieux; BK, Biokit; PM, pacemaker; BNDZ, benznidazole; wk, weeks; PN, peripheral neuropathies; –, normal exam; Fav, favorable.

†Institut Pasteur, cut-off IIF 1/40.

‡Parasitology laboratory, La Pitié Salpétrière hospital, Assistance Publique des Hôpitaux de Paris,(AP-HP) cut-off IIF 1/400; §biological qualification donation laboratory, Etablissement Français du sang, cut-off ELISA Biomérieux 0.9, cut-off ELISA Biokit 0.8; ¶Parasitology laboratory, Université Libre de Bruxelles, cut-off IIF 0.2, cut-off ELISA 1/40.
